# Cross-frequency neuromodulation: leveraging theta-gamma coupling for cognitive rehabilitation in MCI patients

**DOI:** 10.3389/fnagi.2025.1541126

**Published:** 2025-04-30

**Authors:** Xiao Yuan, Zhilan Tu, Renren Li, Chenxi Pan, Jing Ma, Meng Liu, Dan Yang, Hualan Yang, Fangyun Li, Zhi Bie, Yixuan Ku, Yunxia Li

**Affiliations:** ^1^Department of Neurology, Tongji Hospital, School of Medicine, Tongji University, Shanghai, China; ^2^Department of Neurology, Shanghai Pudong Hospital, Fudan University Pudong Medical Center, Shanghai, China; ^3^Department of Neurology, Changhai Hospital, The First Affilitated Hospital of Naval Medical University of PLA, Shanghai, China; ^4^Department of Psychology, Sun Yat-sen University, Guangzhou, China; ^5^Shanghai Key Laboratory of Vascular Lesions Regulation and Remodeling, Shanghai, China

**Keywords:** transcranial magnetic stimulation, theta-gamma coupling, working memory, mild cognitive impairment, cognitive enhancement

## Abstract

**Background:**

Theta-gamma coupling (TGC) plays a critical role in working memory (WM) processing, yet it is often dysregulated in mild cognitive impairment (MCI). While gamma activity is known to support cognitive functions, excessive gamma activity in MCI may impair WM. This study investigates how repetitive transcranial magnetic stimulation (rTMS) modulates gamma activity by regulating TGC to enhance WM in MCI patients.

**Objectives:**

This study aims to explore the effects of rTMS on WM by targeting TGC and reducing occipital gamma activity under varying WM loads.

**Methods:**

We recruited 34 participants, including 20 MCI patients and 14 healthy elderly controls (HC), from Shanghai Tongji Hospital. All participants received a 1 week intervention of 10 Hz rTMS targeting the left dorsolateral prefrontal cortex (DLPFC). Gamma power and TGC were measured using electroencephalography (EEG), and WM performance (accuracy, capacity, reaction time) was assessed through a visual WM paradigm.

**Results:**

The rTMS intervention significantly reduced gamma power in left occipital region, correlating with improved WM performance (enhanced accuracy, capacity, and faster reaction time). Changes in TGC between left frontal theta and occipital gamma oscillations were significantly associated with WM performance, indicating a neurocognitive link.

**Conclusion:**

This study highlights rTMS as a non-invasive tool for enhancing WM by modulating TGC and reducing gamma power. These findings suggest a promising strategy for improving cognitive function in MCI, with significant clinical implications for cognitive optimization and therapeutic interventions.

## 1 Introduction

Mild Cognitive Impairment (MCI) is a critical precursor to more severe neurodegenerative conditions such as Alzheimer’s Disease (AD), with memory impairment being a significant challenge for affected individuals (Petersen et al., 2009). Given that approximately 10%–15% of individuals with MCI progress to AD annually, developing interventions to enhance cognitive function and potentially slow or prevent further decline is of utmost importance (Albert et al., 2011).

Working Memory (WM) is central to the cognitive deficits experienced by individuals with MCI (Kirova et al., 2015). The neurophysiological underpinnings of visuospatial working memory (VWM) involve coordinated activity within and between various brain regions, primarily the prefrontal cortex (PFC), occipital, and parietal cortex (Ku et al., 2015). This coordination is reflected in neural oscillations, specifically theta (4–8 Hz) and gamma (30–80 Hz) rhythms, which are thought to underpin the encoding and retrieval of WM (Lisman and Jensen, 2013). Additionally, theta-gamma phase-amplitude coupling (PAC) has emerged as a critical neural mechanism supporting various cognitive processes (Brooks et al., 2022). In healthy individuals, theta-gamma coupling (TGC) is well-regulated, facilitating efficient cognitive processing (Fernández et al., 2021; Ursino and Pirazzini, 2024). However, in MCI, this coupling is often dysregulated, associated with cognitive dysfunction (Brooks et al., 2020; Canolty and Knight, 2010; Goodman et al., 2018).

Despite the potential of repetitive transcranial magnetic stimulation (rTMS) to enhance cognitive function in patients with MCI, the precise cognitive-neural mechanisms underlying this treatment remain poorly understood (Hu et al., 2024; Jiang et al., 2021; Yan et al., 2023). Prior studies have debated whether gamma activity increases or decreases in specific brain regions (Başar, 2013; Başar et al., 2017; Herrmann and Mecklinger, 2001; Herrmann et al., 2004; Kaiser and Lutzenberger, 2003; Lenz et al., 2008; Rochart et al., 2020; Strüber and Herrmann, 2020, 2022; van Deursen et al., 2008). Additionally, the dynamic changes of TGC throughout various phases of working memory have not been extensively detailed. Our study aims to bridge these gaps by systematically analyzing the changes in gamma activity and TGC before and after rTMS application, thereby advancing our understanding of how precise modulation of neural oscillations can improve cognitive performance in MCI patients.

The study employs a controlled trial involving MCI patients and healthy elderly controls (HCs). Advanced neuroimaging techniques, such as EEG, will be used to monitor changes in neural oscillations and working memory performance before and after the intervention. The primary outcome will be an improvement in WM performance, accompanied by detectable changes in theta-gamma coupling and gamma activity, suggesting underlying neural modifications. The findings of this study could provide valuable insights into the neural mechanisms underlying WM enhancement and offer potential targets for non-invasive brain stimulation techniques aimed at enhancing cognitive function.

## 2 Materials and methods

### 2.1 Participants

Participants for this study were recruited from the Memory Specialist Clinic within the Department of Neurology at Tongji Hospital, affiliated with Tongji University. The study received ethical approval from the Ethics Committee of Tongji Hospital, Shanghai, China, and all participants provided informed consent. MCI diagnosis was based on established criteria (Bondi et al., 2014; Croisile et al., 2012), with participants aged age between 55 and 80 years and having no contraindications for TMS. Exclusion criteria included a history of neurological or psychiatric disorders, significant head trauma, or current use of medications affecting cognitive function. HCs were required to had no memory complaints, exhibit normal cognitive function and demonstrate normal daily living abilities. A total of 50 participants were enrolled, comprising both MCI patients and HCs.

All participants received standardized diagnostic evaluations including computed tomography (CT) or magnetic resonance imaging (MRI) scans of the head, blood tests, and comprehensive neuropsychological assessments. These assessments included memory, language, executive function, visual space navigation function, the Mini-Mental State Examination (MMSE) (Katzman et al., 1988), Montreal Cognitive Assessment (MoCA) (Chen et al., 2016), the Hamilton Depression Rating Scale (HAMD) (Hamilton, 1960), and the Hamilton Anxiety Rating Scale (HAMA) (Hamilton, 1959). Memory function was measured using the Hopkins Verbal Learning Test-Revised (HVLT-R) (Ryan et al., 2021), and the logical memory test (Wechsler memory scale) (Gong et al., 1981). Language function was assessed using the Boston Naming Test (Roth, 2011) and the Verbal Fluency Test (Hart et al., 1988). Executive function was measured using the Shape Trial Test-A and B (STT-A, STT-B) (Zhao et al., 2013). Visual space navigation function was assessed using the Rey-Osterrieth Complex Figure Test (ROCF, including the copy test and the recall test) (Guo et al., 2000).

### 2.2 rTMS procedure

Repetitive transcranial magnetic stimulation was administered using a Pulsed magnetic Stimulation Device (M-100 Ultimate, Shenzhen Yingchi Technology Co.,Ltd, Shenzhen, China) with a 90 mm-figure-of-eight coil. The dorsolateral prefrontal cortex (DLPFC) was targeted based on the Beam F3 system (Beam et al., 2009). The rTMS protocol consisted of high-frequency stimulation at 10 Hz with an intensity of 100% of the resting motor threshold (RMT), Each session delivered a total of 3,000 pulses in 10 s trains with 20 s intertrain intervals. The intervention comprised one daily session lasting approximately 15 min, conducted over a period of 1 week. This optimized protocol balances therapeutic efficacy with safety considerations, adhering to established guidelines for cognitive enhancement research (Cheng et al., 2018; Lin et al., 2019; Liu et al., 2021; Miller et al., 2023). The RMT was defined as the minimum stimulation intensity required to elicit a motor evoked potential (MEP) of at least 50 μV in the contralateral first dorsal interosseous muscle, in at least 5 out of 10 trials (Moscatelli et al., 2023). This standardized approach ensured consistent and accurate stimulation intensity across all participants.

### 2.3 Experimental design

**FIGURE 1:**
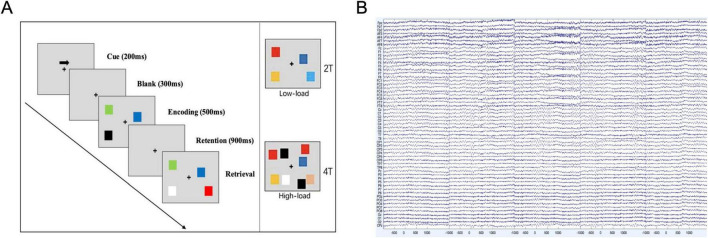
Visual change detection task paradigm and electroencephalography (EEG) recordings. **(A)** Illustrates the procedure of the visual change detection task used to assess visuospatial working memory (VWM) performance, consisting of 100 trials per condition (2 items and 4 items). Participants were directed by an arrow indicating the target hemifield for 200 ms, followed by a 300 ms blank period. A set of colored squares then appeared bilaterally for 500 ms, during which participants were tasked with memorizing the squares in the indicated hemifield. After a 900 ms retention interval, participants responded to a test array by pressing one of two buttons to indicate a match or mismatch with the original set. The test array remained on screen until a response was recorded. **(B)** Displays the raw EEG waveforms synchronized with the task, capturing the electrical activity of the brain throughout the experiment.

Participants were seated in a quiet room, approximately 60 cm away from a computer screen. The VWM task was administered using Eprime 3.0 software, employing an adapted change detection task (see Figure 1; Kyllingsbaek and Bundesen, 2009; Vogel and Machizawa, 2004). Each trial began with a central arrow cue directing participants to memorize items in either the left or right visual hemifield. The EEG data were segmented into three phases based on the timing of the arrow appearance: (1) the Attention phase (1,000–1,500 ms), (2) the Encoding phase (1,500–2,000 ms), and (3) the Retention phase (2,000–3,000 ms). Each participant completed 100 trials per condition [2 items (2T) or 4 items (4T)] in each experiment. The memory array consisted of two colored squares (2T) or four colored squares (4T) presented in each hemifield. Participants were required to indicate whether the test array matched the memory array. Accuracy and mean reaction time were recorded for each participant, and working memory capacity was calculated using Cowan’s K coefficient [K = load × (hit rate - false alarm rate)] (Cowan, 2001). Participants performed the VWM tasks both before and after the 1 week rTMS intervention to assess changes in performance.

### 2.4 EEG recording and PSD analysis

Electroencephalography data were recorded using a 64-channel portable wireless EEG system (Neuracle, Changzhou, China), and 64-channel Brain Products EEG system (Germany). Data were preprocessed offline using EEGLAB 2020 and custom MATLAB 2017. EEG signals are first segmented into 2 s intervals, and any segment exceeding five times the average intensity is automatically identified as an artifact and removed based on the predefined threshold. The subsequent processing included band-pass filtering (0.5–95 Hz), notch filtering at 50 and 100 Hz to remove power line noise, and artifact removal via independent component analysis (ICA) targeting eye blinks, muscle activity, heartbeats, and channel noise. The data were then segmented into epochs from 1,000 ms pre-stimulus to 2,000 ms post-stimulus, with an artifact rejection threshold set at ± 100 μV. The study utilized the standard frequency bands as follows: delta (0.5–4 Hz), theta (4–8 Hz), alpha (8–12 Hz), beta (12–30 Hz), low-gamma (30–60 Hz), and high-gamma (60–80 Hz). Power spectral density (PSD) for these bands was calculated using the fast Fourier transform (FFT) on 2 s segments of EEG signals, providing a frequency resolution of 0.5 Hz.


F⁢(ω)=f⁢f⁢t⁢(X)

$$P\,o\,w\,e\,r_{b\,a\,n\,d}=\sum_{\mathrm{lower}\,\mathrm{bound}}^{\mathrm{upper}\,\mathrm{bound}}{\left|F\,\left(\omega \right)\right|}^{2}$$

Brain regions were divided according to the following electrode allocations: left frontal: Fp1, AF3, AF7, F1, F3, F5, F7, FC1, FC3, FC5; right frontal: Fp2, AF4, AF8, F2, F4, F6, F8, FC2, FC4, FC6; left central: C1, C3, C5, CP1, CP3, CP5; right central: C2, C4, C6, CP2, CP4, CP6; left temporal: FT7, T7, TP7; right temporal: FT8, T8, TP8; left occipital: P3, P5, P7, PO3, PO7, O1; right occipital: P4, P6, P8, PO4, PO8, O2.

### 2.5 Cross-frequency coupling analysis

This systematic categorization enabled a detailed examination of neural activity across different brain areas. Theta-gamma PAC was calculated using the modulation index method (Tort et al., 2010), and phase-phase coupling (PPC) were also computed. The Hilbert transform was applied to extract the instantaneous phase of theta oscillations (4–8 Hz) and the amplitude envelope of gamma oscillations (30–80 Hz). The modulation index (MI) was computed to quantify PAC between theta phase and gamma amplitude (Tort et al., 2010), The MI quantifies PAC by measuring the deviation of the amplitude distribution from uniformity when binned according to phase. Specifically, the phase was divided into 18 bins (20° each), and the amplitude within each bin formed a distribution.


$$\text{MI}=\frac{1}{\text{log}\,\left(N\right)}\,\sum_{i=1}^{N}P_{i}\,\text{log}\,\left(\frac{P_{i}}{1/n}\right)$$

where N is the number of phase bins, and P_i_is the normalized amplitude within the *i*-th bin. This index quantifies the degree of deviation from a uniform distribution, with higher values indicating stronger coupling. The MI reflects the deviation of the amplitude distribution from uniformity, serving as a measure of coupling strength.

### 2.6 Statistical analysis

All analyses were conducted with SPSS (version 29.0) and RStudio (version 4.1.3). For continuous variables, either the Mann–Whitney U-test (for non-normally distributed data) or Student’s *t*-test (for normally distributed data) was applied, depending on the data distribution. Categorical variables were analyzed using Pearson’s χ^2^ test. Spearman correlation analyses were performed to explore relationships between occipital power or TGC and VWM performance. Changes in WM performance, PSD, and TGC following rTMS intervention were evaluated using paired *t*-tests. Statistical significance was set at *P* < 0.05. To control for multiple comparisons, False Discovery Rate (FDR) correction was applied using the Benjamini-Hochberg procedure, with corrected *p*-values <0.05 considered statistically significant (see as Supplementary Tables 1, 2).

## 3 Results

### 3.1 Baseline demographic and neuropsychological characteristics

The study included 34 participants, comprising 20 MCIs and 14 HCs (See as Supplementary Figure 1). As detailed in Table 1, the MCI and HC groups were well-matched in terms of demographic characteristics including age, gender, education, as well as emotional status assessed by HAMA and HAMD (all *P* > 0.05). As expected, the MCI group exhibited significantly lower scores in global cognitive performance, as measured by MMSE and MoCA (both *P* < 0.001). Additionally, impairments were observed across specific cognitive domains, including memory (HVLT_IR, HVLT_DR and LMT scores; *P* < 0.001), executive function (STT-A scores; *P* < 0.01), visuospatial ability (ROCF_DR scores; *P* < 0.01), and language (BNT and VFT scores; *P* < 0.01). The demographic and emotional equivalence between the groups ensures a balanced baseline, minimizing the influence of confounding variables and enhancing the reliability of the study design.

**TABLE 1:** Patient demographics and baseline characteristics.

Characteristic	HC *n* = 14	MCI *n* = 20	*P*
Age	68 (57, 76)	65 (63, 73)	0.726^a^
Gender, female (%)	6 (42.9%)	10 (50.0%)	0.681^c^
Education	12.0 (9.5, 14.3)	12.0 (9.0, 16.0)	0.887^a^
MMSE	28.0 (28.00, 28.75)	25.5 (24.00, 26.25)	**< 0.001** *** ^a^
MoCA	23.9 ± 3.1	17.2 ± 4.2	**< 0.001** *** ^b^
HAMA	6.0 (3.3, 10.0)	6.5 (4.8, 11.0)	0.806^a^
HAMD	4.5 (1.5, 7.0)	6.0 (3.0, 8.3)	0.471^a^
HVLT_IR	7.95 ± 1.51	4.92 ± 1.55	**< 0.001** *** ^b^
HVLT_DR	7.5 (7.0, 9.8)	3.0 (0.0, 5.0)	**< 0.001** *** ^a^
LMT	10.21 ± 2.12	6.25 ± 2.71	**< 0.001** *** ^b^
BNT	24.5 (23.0, 27.0)	20.0 (15.8, 24.3)	**0.009** ** ^a^
VFT	14.4 ± 3.5	10.3 ± 3.7	**0.002** ** ^b^
STT-A	48 (40, 54)	71 (52, 95)	**0.003** ** ^a^
STT-B	120 (105, 146)	153 (121, 223)	0.064^a^
ROCF	35.5 (35.0, 36.0)	32.5 (30.0, 36.0)	0.118^a^
ROCF_DR	19 (16, 29)	9 (2, 14)	**0.004** ** ^a^

^a^Wilcoxon rank sum test.

^b^Independent Two Sample *t*-test.

^c^Pearson’s Chi-squared test; Statistical significance evidenced in bold. Values are Median (IQR); Mean ± SD; BNT, Boston Naming Test; HAMA, Hamilton Anxiety Rating Scale; HAMD, Hamilton Depression Rating Scale; HVLT_DR, Hopkins Verbal Learning Test-Delayed Recall; HVLT_IR, Hopkins Verbal Learning Test-Immediate Recall; LMT, Logical Memory Test; MMSE, Mini-Mental State Examination; MoCA, Montreal Cognitive Assessment; ROCF, Rey-Osterrieth Complex Figure Test; ROCF_DR, Rey-Osterrieth Complex Figure Test-Delayed Recall; STT-A, Shape Trail Test-A; STT-B, Shape Trail Test-B; VFT, Verbal Fluency Test.

Statistical significance: *p < 0.05, **p < 0.01, ***p < 0.001.

### 3.2 Working memory performance post-TMS intervention

**FIGURE 2:**
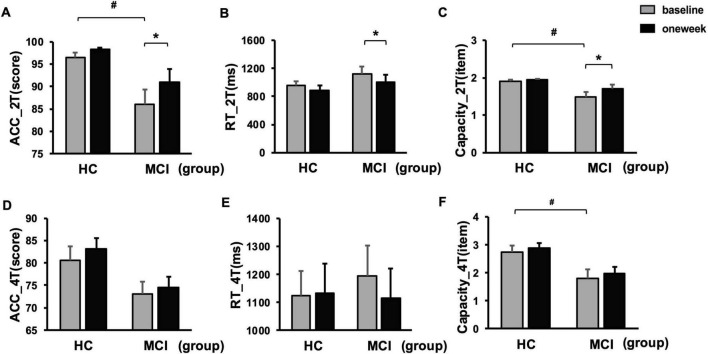
Changes in visuospatial working memory (VWM) performances between MCI and HC. **(A–C)** Mean working memory performance in terms of accuracy, reaction time and capacity under low memory load (2T) before and after rTMS intervention. **(D–F)** Mean accuracy, reaction time and capacity under high memory load (4T) before and after rTMS intervention. Error bar represented standard error. Paired *t*-tests, *p < 0.05, pre vs. post; two-sample *t*-tests, ^#^*p* < 0.05, MCI vs HC. 2T, 2-items; 4T, 4-items; ACC, accuracy; HC, healthy controls; MCI, mild cognitive impairment; RT, reaction time.

Prior to the intervention, under low memory load (2T), the MCI group exhibited significantly lower working memory accuracy and capacity compared to the HC group. Following the 1 week TMS intervention, the MCI group demonstrated significant improvements in working memory performance, as evidenced by increased accuracy, enhanced capacity, and faster reaction times (*P* < 0.05; see Figure 2). However, there were no statistical differences under high WM load (4T).

### 3.3 Neural changes during low WM load (2T)

**FIGURE 3:**
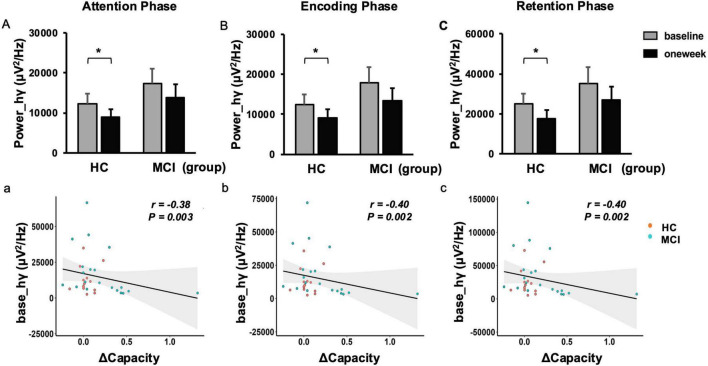
Changes in spectral power and memory capacity correlation for 2-items (2T). Absolute band power reduced at the high-gamma frequency in the left occipital region during the attention phase **(A)**, the encoding phase **(B)**, and the retention phase **(C)** following rTMS. The negative correlation between increased memory capacity and baseline high gamma power was also assessed for the attention phase **(a)**, the encoding phase **(b)**, and the retention phase **(c)**. Error bar represented standard error. Paired *t*-test,*p < 0.05, pre vs. post. ΔCapacity, change in memory capacity post- and pre-rTMS; hγ, high-gamma; MCI, mild cognitive impairment; rTMS, repetitive transcranial magnetic stimulation.

Our study investigated the effects of rTMS on occipital gamma power during WM tasks. Importantly, there were no significant baseline differences between MCI and HC groups in either gamma power or theta-gamma coupling (both *p* > 0.05). The findings revealed a consistent and significant reduction in left occipital gamma power in HCs across WM phases following rTMS administration (see Figures 3A–C). In contrast, patients with MCIs demonstrated only a non-significant trend toward reduced gamma power.

Repetitive transcranial magnetic stimulation significantly modulated TGC between the left frontal theta and left occipital gamma oscillations under low WM load condition. Specifically, in individuals with MCI, we observed a significant increase in TGC during the attention phase and a significant decrease during the retention phase (both *P* < 0.05; see Figures 5A, C; Supplementary Figures 2A,a,C,c).

Further correlation analysis revealed significant relationships between rTMS-induced neural alterations and behavioral performance. Notably, the enhancement of left occipital gamma power throughout the WM process was negatively correlated with improvements in memory capacity (both *P* < 0.05; see Figures 3a–c). Moreover, the negative correlation between TGC in the retention phase and WM accuracy highlights the crucial role of neural synchronization in cognitive processing (*P* < 0.05; see Figure 5D).

### 3.4 Neural changes during high WM load (4T)

**FIGURE 4:**
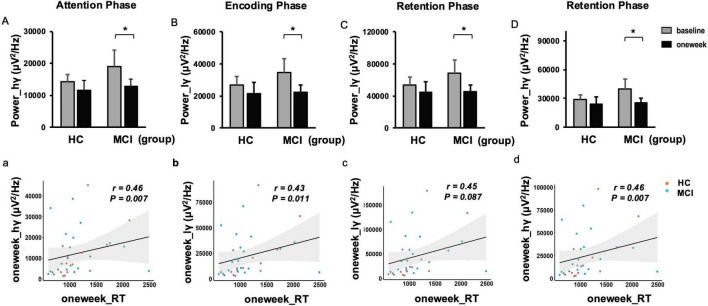
Changes in spectral power and reaction time correlation for 4-items (4T). Absolute band power reduced at high gamma frequency in the left occipital region post repetitive transcranial magnetic stimulation (rTMS) **(A)** and its positive correlation with 1 week reaction time **(a)** during the attention phase. Low gamma power reduced **(B)** and its positive correlation with 1 week reaction time **(b)** during the encoding phase. Low gamma **(C)** and high gamma **(D)** power reduced, and, respectively their positive correlations with 1 week reaction time **(c,d)** during the retention phase. Error bar represented standard error. Paired *t*-test, *p < 0.05, pre vs. post. hγ, high-gamma; lγ, low-gamma.

Under high WM load, there were no significant baseline differences between MCI and HC groups in gamma power or theta-gamma coupling (both *p* > 0.05). However, our findings demonstrated a significant decrease in gamma power within the left occipital region throughout the WM process following rTMS in MCI patients (both *P* < 0.05; see Figures 4A–D). This reduction in occipital gamma power was positively correlated with faster reaction time, indicating improved processing speed (both *P* < 0.05; see Figures 4a–d).

**FIGURE 5:**
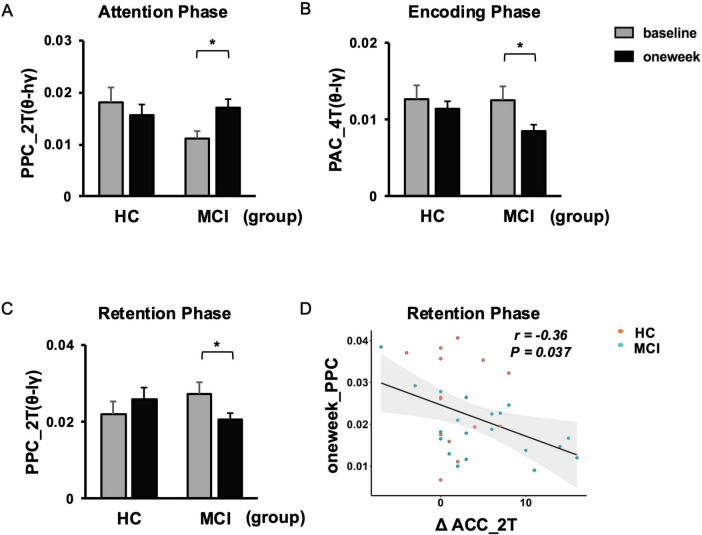
Changes in TGC under working memory (WM) loads and correlation with WM performances. Under low WM load (2T), the degree of PPC increased between left frontal theta and left occipital high gamma oscillations during the attention phase **(A)**. Under high WM load (4T), the degree of PAC decreased between left frontal theta and left occipital low gamma oscillations during the encoding phase **(B)**. Under low WM load, the degree of PPC decreased between left frontal theta and left occipital low gamma oscillations during the retention phase **(C)**, along with the negative correlation between the 1 week PPC and the increased accuracy **(D)**. Error bar represented standard error. Paired *t*-test, *p < 0.05, pre vs. post. ΔACC, change in memory accuracy post- and pre-rTMS; PAC, phase-amplitude coupling; PPC, phase-phase coupling; TGC, theta-gamma coupling.

Furthermore, our study explored the effects of rTMS on the coupling between left frontal theta and left occipital gamma oscillations under high WM load condition. A significant decrease in coupling was observed during the encoding phase *P* < 0.05; see Figure 5B; Supplementary Figures 2B,b.

## 4 Discussion

In this study, rTMS enhanced working memory in MCI patients by improving accuracy, capacity, and reaction time under low WM load but showed no significant effects under high WM load. The neural mechanisms underlying these effects appear to involve rTMS-induced modulation of neural oscillations, specifically through increased TGC during the attention phase and decreased TGC during the retention phase under low memory load condition. Interestingly, while high memory load condition did not yield significant performance improvements, we observed a notable reduction in TGC during the encoding phase. This suggests that the therapeutic efficacy of rTMS may be constrained by task complexity, potentially due to the increased neural resource demands associated with high cognitive load.

### 4.1 Mechanisms and efficacy of TMS in modulating neural oscillations

#### 4.1.1 Improvements in working memory post-rTMS

The improvements in working memory after rTMS are significant, supporting studies that highlight TMS as a tool for enhancing cognitive functions in MCI (Licht et al., 2023). Zhang et al. (2022) demonstrated notable cognitive gains in MCI patients, particularly in tasks involving working memory and executive function. This suggests that TMS may improve cognitive processing by modulating neural circuits involved in working memory.

The cognitive-enhancing effects of TMS in low working memory load are mediated through multiple neurophysiological mechanisms. The findings of this study demonstrates that TMS optimizes information processing efficiency, as evidenced by improved accuracy, capacity, and reaction time These improvements primarily depend on the short-term potentiation of local oscillatory synchronization (Salimpour and Anderson, 2024) and synaptic plasticity, which are fundamental processes underlying cognitive enhancement (Hallett, 2007; Licht et al., 2023; Riddle et al., 2022). Specifically, the enhancement of theta-slow gamma coupling reflects a compensatory mechanism that maintains spatial working memory performance under increasing task demands (Tamura et al., 2017). Furthermore, neuroimaging studies have shown that TMS modulates functional connectivity within key brain networks, particularly the frontoparietal network and default mode network, which are critically involved in working memory processes (Johnson et al., 2024).

However, under high cognitive load, the regulatory efficacy could be constrained by the saturation of neural resources, increased competition, and imbalanced resource allocation. While intracranial EEG reveals that TCG activation scales with cognitive difficulty in epilepsy patients (Axmacher et al., 2010), MCI patients exhibit consistent underperformance across cognitive tasks compared to healthy controls (Rosano et al., 2005). This discrepancy suggests that, although significant neural changes are observed in MCI patients, these changes may not be sufficient to overcome the neural resource limitations imposed by high cognitive load. Consequently, these alterations fail to reach the threshold necessary for translating into measurable behavioral improvements.

Moreover, high-load tasks necessitate coordinated cross-frequency coupling across multiple bands. TMS modulation of θ-γ rhythms may disrupt natural frequency interactions, particularly α-γ coupling’s critical role in suppressing irrelevant information (Roux and Uhlhaas, 2014). Left DLPFC stimulation could impair resource allocation, potentially disrupting working memory processes (Moss et al., 2004). This may reduce errors for low-priority items without enhancing high-priority performance, ultimately limiting overall efficacy (Viejo-Sobera et al., 2017). Distinct populations demonstrate varied cognitive strategies under high demands (Yagura et al., 2024), highlighting the complexity of neural regulation mechanisms.

#### 4.1.2 Occipital gamma changes associated with rTMS

The nature of gamma activity alterations in AD remains controversial (Strüber and Herrmann, 2022). While some EEG studies report increased gamma activity (Başar, 2013; Başar et al., 2017; Rochart et al., 2020; van Deursen et al., 2008) others demonstrate decreased gamma oscillations (Herrmann and Mecklinger, 2001; Herrmann et al., 2004; Kaiser and Lutzenberger, 2003; Lenz et al., 2008; Strüber and Herrmann, 2020). This discrepancy likely stems from methodological variations in brain regions, task paradigms, and EEG recording conditions (resting-state vs. task performance).

Neuroimaging evidence suggests increased activation in the posterior cingulate and medial parietal cortices during task performance in AD patients (Lustig et al., 2003), though the underlying oscillatory mechanisms remain unclear. Recent EEG findings provide further insights: older adults with amyloid and tau pathology exhibit elevated low-gamma activity in frontal-central regions during low-load working memory tasks, potentially indicating compensatory mechanisms under reduced cognitive demand (Blanco-Duque et al., 2024). In AD patients, target detection tasks with simple visual stimuli reveal prolonged reaction times accompanied by abnormal gamma increases (Başar, 2013; Başar et al., 2017). Our findings corroborate these observations, demonstrating a positive correlation between reaction time and left occipital gamma activity during working memory tasks, suggesting a central role of gamma dysregulation in cognitive impairment.

The distinct gamma modulation patterns observed between HCs and MCI patients across varying memory load conditions may reflect fundamentally different neural compensation mechanisms. In HCs, the reduction of gamma activity under low memory load following rTMS intervention suggests an optimization of neural resource allocation, indicating enhanced neural efficiency (Zhao et al., 2017). Similarly, in MCI patients, the observed gamma reduction under high memory load may reflect rTMS-mediated regulation of compensatory over activation, potentially restoring more efficient oscillatory patterns during cognitively demanding tasks, thereby making the regulatory effects of rTMS more pronounced. This load-dependent and population-specific response pattern likely originates from differential neural network reorganization in MCI patients, contrasting with the preserved neural efficiency in HCs, underscoring the complex neurophysiological mechanisms that underlie rTMS-induced cognitive enhancement.

#### 4.1.3 Theta-gamma changes associated with rTMS

The significant neural changes observed, particularly the modulation of TGC by rTMS, offer key insights into the cognitive improvements seen in MCI patients. TGC is crucial for cognitive processes like attention, memory, and executive function (Goodman et al., 2018), aiding in the efficient processing and integration of information (Ahn et al., 2022; Buzsáki and Draguhn, 2004; Lisman and Jensen, 2013). Disruption of theta-gamma coupling in MCI may contribute to deficits in memory and executive function (Gazzaley and Nobre, 2012; Goodman et al., 2018).

Our findings reveal distinct TGC dynamics across cognitive phases and memory loads. During low working memory load conditions, TGC increases during the attention phase. This phase-specific modulation aligns with TGC’s functional roles: enhanced TGC during attention facilitates neural synchronization for stimulus focus and encoding improvement (Kim et al., 2016), where selective attention determines information maintenance and processing (Mirjalili et al., 2022; Rajji et al., 2016). The decrease during retention may reflect a shift in neural dynamics that optimizes maintain encoded information by minimizing interference from extraneous neural activity.

Under high working memory load, we observed decreased TGC during encoding, particularly in cognitive control networks such as the DLPFC. This reduction likely reflects the brain’s resource allocation strategy, where limited neural resources are prioritized for essential processing demands (Brzezicka et al., 2019; Funahashi, 2017). The DLPFC, crucial for information management and manipulation, appears particularly sensitive to these load-dependent changes. The application of rTMS to the DLPFC may modulate its functional connectivity with downstream regions (e.g., parietal and occipital cortices), reducing excessive or unnecessary neural synchronization (Khan et al., 2023). This modulation could enhance the precision and efficiency of information encoding, ultimately optimizing cognitive performance under high-load conditions.

Recent advances in neuroimaging have elucidated TGC’s dual role in local processing and long-range cortical communication (Boyd et al., 2007; Tavano et al., 2023; Zeng et al., 2022). The demonstrated ability of TMS to modulate TGC underscores its therapeutic potential for MCI. By targeting and potentially restoring disrupted oscillatory dynamics, TMS may offer a promising approach to mitigate cognitive decline through neural network modulation.

#### 4.2 Other cross-frequency coupling mechanisms in working memory

Beyond theta-gamma coupling, other cross-frequency interactions play crucial roles in working memory processes. Alpha-gamma PAC mediates information selection through cyclic inhibition of gamma activity (> 25 Hz) by alpha oscillations (8–12 Hz), with brief gamma disinhibition during alpha troughs facilitating task-relevant information processing (Bonnefond and Jensen, 2015). This coupling mechanism, predominantly observed in parietal-prefrontal networks, serves critical functions in suppressing irrelevant sensory inputs and enhancing multimodal integration in visual and verbal working memory (de Vries et al., 2020; Popov et al., 2018). Experimental evidence demonstrates a positive correlation between alpha-gamma PAC strength and memory load (Yagura et al., 2024), with gamma-tACS showing potential to selectively enhance memory accuracy (Thompson et al., 2021). The experimental results underscore the critical role of alpha-gamma phase synchronization in mediating neural mechanisms underlying memory formation, attentional control, and higher-order cognitive functions, particularly in terms of task-specific neural dynamics and working memory content-dependent oscillatory patterns (Yagura et al., 2024).

Theta-beta coupling (4–8 and 13–30 Hz) supports sequential information storage through temporal organization of beta activity, while theta oscillations modulate prefrontal beta activity to enhance executive control (Benchenane et al., 2011). Research indicates that prefrontal theta power increases with working memory load, whereas beta activity may maintain task-set stability, with both rhythms coordinating spatial memory and hippocampal-prefrontal synchronization. The age-related decrease in prefrontal theta-beta ratio (TBR) during childhood and adolescence reflects the maturation of attentional control (Chen et al., 2023). Emerging interventions, including neurofeedback and theta-tACS, demonstrate potential in modulating TBR to improve executive functions (Arnold et al., 2013). Further evidence of frequency-specific cognitive adaptation comes from beta-band phase-amplitude coupling studies, which demonstrate an inverse relationship between coupling intensity and arithmetic task complexity (Daume et al., 2017). This finding suggests a dynamic, task-dependent reorganization of neural oscillations to optimize cognitive processing.

### 4.3 Limitations and future directions

While this study makes important contributions to understanding rTMS-induced modulation of TGC and its behavioral correlates, several limitations should be noted. First, the relatively small sample size may limit the generalizability of our findings and increase the risk of Type II errors, underscoring the need for larger-scale studies with longer-term follow-up to evaluate the durability of both cognitive and neural improvements. More importantly, while our findings demonstrate a significant association between rTMS-induced theta-gamma coupling changes and working memory improvement, the lack of sham control in MCI participants and inability to determine oscillatory directionality limit causal interpretation. Future studies should combine active sham protocols with directional connectivity analyses to both verify the rTMS-specific nature of these effects and elucidate whether frontal theta drives occipital gamma modulation during working memory processes.

Additionally, the study primarily relied on visual WM tasks and theta-gamma coupling as neural outcomes, which may not capture the full range of TMS effects. Future research should incorporate additional neural markers (e.g., alpha oscillations, connectivity patterns) and multimodal paradigms to validate the generalizability of findings across different WM domains.

Finally, while this study demonstrates TMS’s potential for enhancing cognition in MCI while revealing significant individual variability in treatment response, which may stem from both the limitations of standardized Beam F3 targeting (Herwig et al., 2003) and the current lack of AD pathology biomarkers in our study design. Future studies employing MRI-guided neuronavigation for improved targeting precision, combined with biomarker assessment (e.g., p-tau217, neurofilament light) for better patient stratification and machine learning approaches integrating multimodal data, could significantly advance the development of personalized TMS protocols for neurodegenerative disorders.

## 5 Conclusion

This study demonstrates TMS’s efficacy in enhancing working memory in MCI patients, particularly under low cognitive load, through phase-specific modulation of theta-gamma coupling and occipital gamma power, revealing novel neural mechanisms of cognitive improvement. These findings not only advance our understanding of non-invasive brain stimulation but also highlight neural oscillations as promising therapeutic targets. Future research should continue to explore the mechanisms underlying these effects, as well as the long-term and individualized impacts of TMS on cognitive function.

## Data Availability

The raw data supporting the conclusions of this article will be made available by the authors, without undue reservation.
