# Supplementary material for: Cross-frequency neuromodulation: leveraging theta-gamma coupling for cognitive rehabilitation in MCI patients
Source: Front Aging Neurosci. 2025 Apr 30;17:1541126. doi: 10.3389/fnagi.2025.1541126 (PMC12075380; doi:10.3389/fnagi.2025.1541126)
Supplement: Supplementary file 1 [file Data_Sheet_1.docx]

**Supplementary material**

**
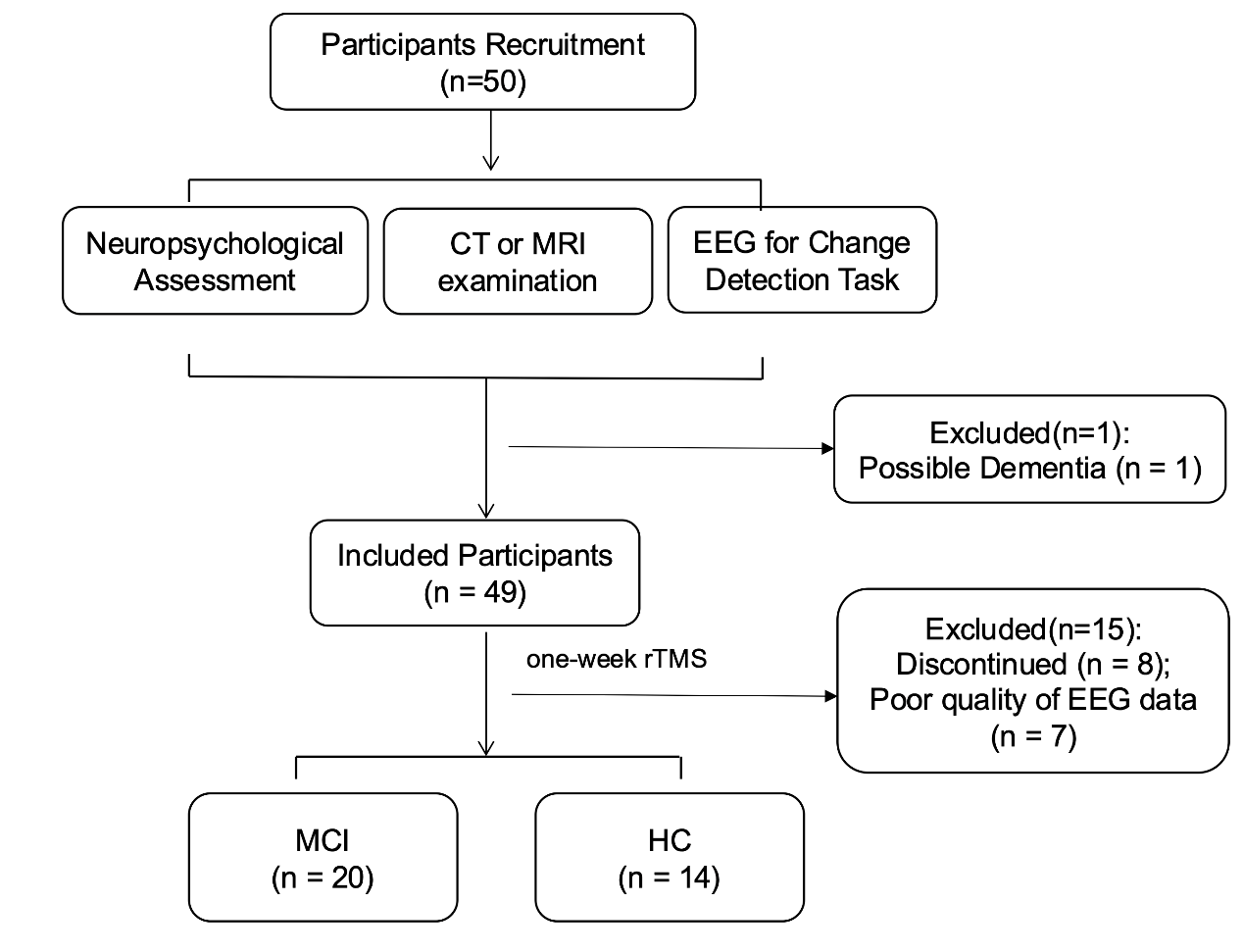
**

**Fig S1 Flow chart of the study**


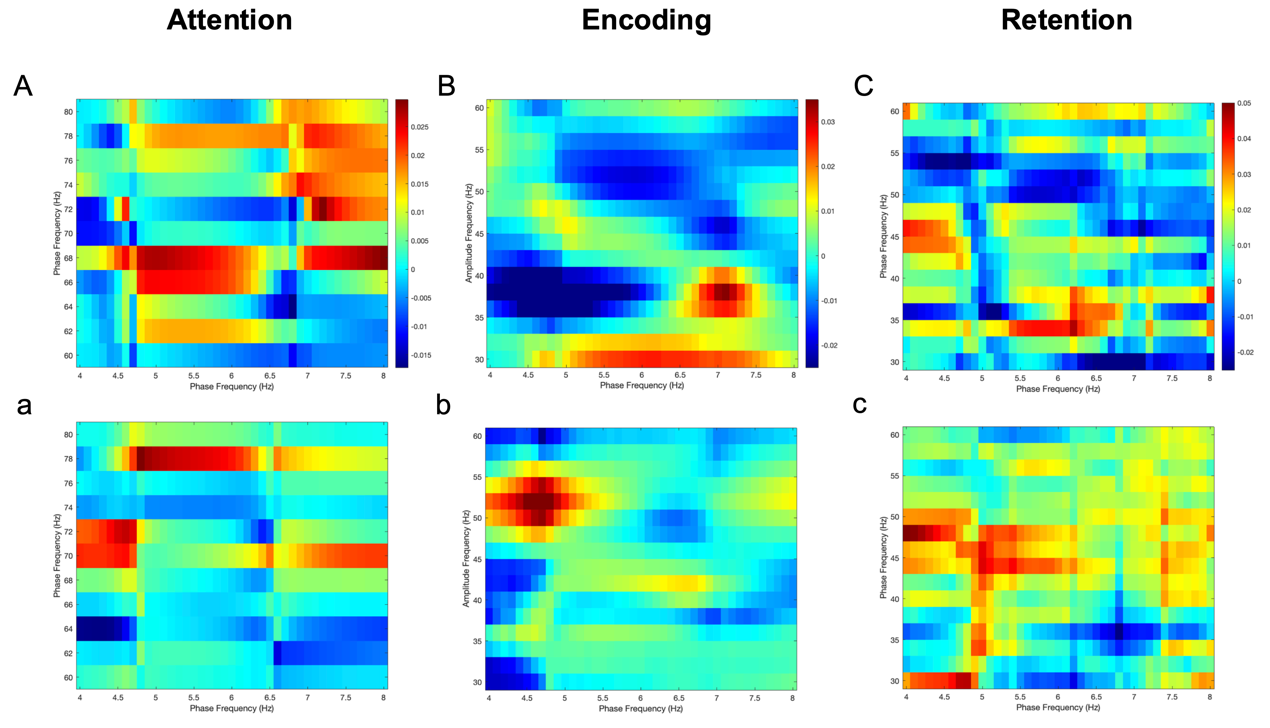
**Fig S2 TMS-Induced CFC Changes in MCI vs. HC**

This figure highlights significant results corresponding to Figure 5, showcasing the changes in CFC following TMS in both MCI and HC groups during specific phase of VWM. (A–C) CFC differences (post- minus pre-stimulation) in the MCI group during the attention phase under 2T load, the encoding phase under 4T load, and the retention phase under 2T load, respectively. (a–c) Corresponding CFC changes in the HC group during the same phase. The heatmaps visually represent the differences in CFC between the two groups before and after stimulation across significant stages and frequency bands. The x-axis and y-axis indicate frequency (Hz), and the color bar denotes changes in coupling intensity, with red and blue denoting increased and decreased coupling intensity, respectively

Table S1: Results of gamma power during different phases of VWM

| **Load** | **Phase** | **HC-pre** | | **HC-post** | ***t*** | ***P*** |  | **MCI-pre** | **MCI-post** | ***t*** | ***P*** |
| --- | --- | --- | --- | --- | --- | --- | --- | --- | --- | --- | --- |
| **2T** | **Attention**  **(**hγ**)** | | 12,310  (9,314) | 8,951 (7,651) | 2.34 | **0.036*** |  | 17,314 (17,043) | 13,857 (14,429) | 1.44 | 0.167 |
|  | **Encoding**  **(**hγ**)** | | 12,515 (9,428) | 9,061 (8,219) | 2.38 | **0.036*** |  | 17,805 (18,214) | 13,416 (13,958) | 1.72 | 0.102 |
|  | **Retention**  **(**hγ**)** | | 25,021 (19,264) | 17,733 (15,224) | 2.55 | **0.036*** |  | 35,199 (36,050) | 27,201 (28,508) | 1.61 | 0.125 |
| **4T** | **Attention**  **(**hγ**)** | | 14,221  (8,889) | 11,465 (12,272) | 0.79 | 0.443 |  | 19,041  (22,342) | 12,707  (10,860) | 2.13 | **0.047*** |
|  | **Encoding**  **(**lγ**)** | | 26,976  (19,903) | 21,656  (25,439) | 0.68 | 0.509 |  | 34,810  (38,110) | 22,448  (18,665) | 2.44 | **0.047*** |
|  | **Retention**  **(**lγ**)** | | 53,628  (37,081) | 44,310  (51,534) | 0.61 | 0.553 |  | 68,233  (75,250) | 45,431  (37,205) | 2.22 | **0.047*** |
|  | **Retention**  **(**hγ**)** | | 29,024  (17,739) | 24,263  (27,690) | 0.63 | 0.538 |  | 39,558  (47,736) | 25,603  (22,081) | 2.12 | **0.047*** |

Note: Values in brackets indicate standard deviation. Paired t-tests were used to compare pre- and post-intervention values. FDR correction was applied to significant results to control for multiple comparisons, with *p < 0.05 and statistical significance evidenced in bold.

Abbreviations: hγ, high-gamma; lγ, low-gamma.

Table S2: Results of PAC/PPC during different phases of VWM

| **Load** | **Phase** | **HC-pre** | | **HC-post** | ***t*** | ***P*** |  | **MCI-pre** | **MCI-post** | ***t*** | ***P*** |
| --- | --- | --- | --- | --- | --- | --- | --- | --- | --- | --- | --- |
| **2T** | **Attention** | | 0.018  (0.011) | 0.016  (0.008) | -0.69 | 0.497 |  | 0.012  (0.007) | 0.016  (0.007) | 2.20 | **0.046*** |
| **4T** | **Encoding** | | 0.013  (0.007) | 0.011  (0.004) | -0.56 | 0.583 |  | 0.014  (0.007) | 0.010  (0.004) | -2.07 | **0.046*** |
| **2T** | **Retention** | | 0.022  (0.012) | 0.026  (0.011) | 0.90 | 0.379 |  | 0.027  (0.013) | 0.019  (0.007) | -2.16 | **0.046*** |

Note: Values in brackets indicate standard deviation. Paired t-tests were used to compare pre- and post-intervention values. FDR correction was applied to significant results to control for multiple comparisons, with *p < 0.05 and statistical significance evidenced in bold.
